# Nearly Complete Genome Sequences of 17 Enterovirus D68 Strains from Kansas City, Missouri, 2018

**DOI:** 10.1128/MRA.00388-19

**Published:** 2019-11-07

**Authors:** Suman B. Pakala, Yi Tan, Ferdaus Hassan, Annie Mai, Robert H. Markowitz, Meghan H. Shilts, Seesandra V. Rajagopala, Rangaraj Selvarangan, Suman R. Das

**Affiliations:** a Department of Medicine, Vanderbilt University Medical Center, Nashville, Tennessee, USA; b Department of Pathology and Laboratory Medicine, Children’s Mercy, Kansas City, Missouri, USA; c University of Missouri-Kansas City School of Medicine, Kansas City, Missouri, USA; d Department of Pathology, Microbiology, and Immunology, Vanderbilt University School of Medicine, Nashville, Tennessee, USA; KU Leuven

## Abstract

Here, we report 17 nearly complete genome sequences of enterovirus D68 (EV-D68) isolated from Kansas City, MO, in 2018. Phylogenetic analysis suggests that these strains belong to subclade B3, similar to the ones that caused the 2016 epidemics in the United States but different from the 2014 outbreak B1 strains.

## ANNOUNCEMENT

**FIG 1:**
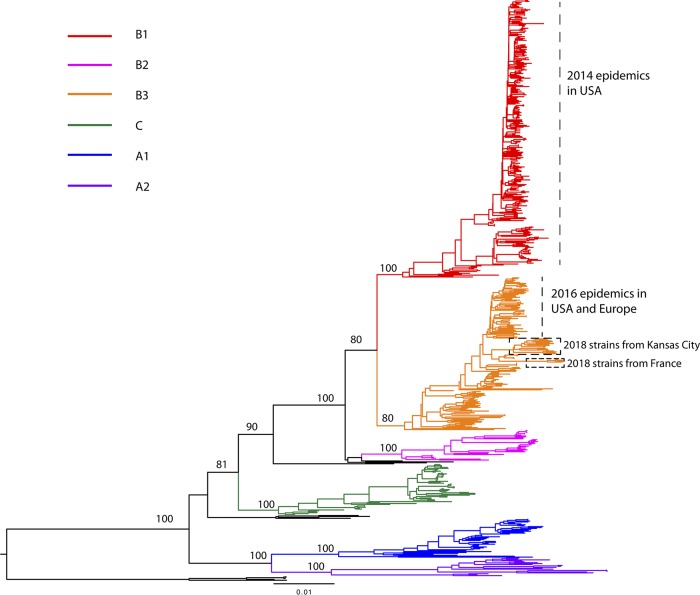
We combined the 17 newly acquired complete EV-D68 genome sequences collected in 2018 in Kansas City with EV-D68 genome sequences available in GenBank (as of 31 January 2019) and generated a data set with a total of 628 complete genome sequences of EV-D68. Sequences were aligned using the MUSCLE program in MEGA6, with manual adjustments (15). The global phylogenetic tree of EV-D68 was inferred using the neighbor-joining method conducted in MEGA6, with the Kimura 2-parameter test and bootstrap test of 1,000 replicates (16). The tree is midpoint rooted and drawn to scale, with branch lengths in the same units as those of the evolutionary distances used to infer the phylogenetic tree. Well-supported nodes by bootstrap values over 70% are shown next to the branches. Six major clades/subclades (B1, B2, B3, C, A1, and A2) are described in the tree. New 2018 sequences sampled from Kansas City are marked in the tree as well.

Enterovirus D68 (EV-D68) belongs to the genus *Enterovirus* in the family *Picornaviridae*, with a single positive-strand RNA genome of ∼7.5 kb in length coding for 4 structural proteins (VP1 to VP4) and 7 nonstructural proteins (2A to 2C and 3A to 3D) (1). EV-D68 was first isolated in California in 1962 from patients with bronchiolitis and pneumonia (2). Between 1962 and 2014, EV-D68 was sporadically detected in different parts of the world (3, 4). However, in August 2014, a new subclade of EV-D68, B1, caused a nationwide outbreak in the United States and an increased number of acute flaccid myelitis (AFM) cases (5, 6). In 2016, a different subclade of EV-D68 strains, B3, spread in some states, such as New York and Missouri, and caused local epidemics (Fig. 1) (7, 8). EV-D68 continues to spread sporadically and was reported in Europe, with more AFM cases involved, in 2018 (9, 10).

Previously, we had developed a high-throughput complete genome sequencing pipeline for EV-D68 to describe the early outbreak of EV-D68 in Kansas City, MO, in 2014 (11). Here, we report the genome sequence of EV-D68, which was obtained from 17 patients from Kansas City between August and October 2018. EV-D68 was confirmed by quantitative PCR (qPCR) (12). After RNA extraction, full-length cDNA was reverse transcribed with the first-strand synthesis SuperMix kit. Two overlapping amplicons (a small [S] 904-bp and large [L] 6.8-kbp amplicon) were generated using EV-D68-specific primers, as described before (11). Libraries were constructed with the NEBNext Ultra II FS DNA kit, samples were pooled, and sequencing was performed using an Illumina MiSeq instrument with 2 × 250-bp reads.

The median number of reads generated per sample was 376,488 (interquartile range [IQR], 326,864 to 421,360 reads). Sequencing reads were binned by barcode, adapters were trimmed with Cutadapt (v 1.18) (13), and low-quality bases were removed with Trimmomatic (v 0.36) (14). Reads were normalized using BBTools (v 38.34) (14) and initially assembled *de novo* using the SPAdes assembler (v 3.13.0) (15), using default parameters. BLASTN searches of the resulting contigs identified an EV-D68 isolate from 2016, with GenBank accession number KY385889 (enterovirus D68 isolate NY212_16, complete genome), as the closest match. Using this sequence as a reference, consensus sequences were produced for all 17 samples using CLC Genomics Workbench (v 11.0.1), with length and similarity fraction thresholds set at 0.9. The average depth of coverage varied between 224× and 4,228×. All 17 sequences are 7,331 bp in length, and their G+C contents vary between 41.6% and 41.9%. They were annotated using the VAPiD annotation pipeline (v 1.6.2) (16) and submitted to GenBank.

Although these 17 2018 EV-D68 isolates from Kansas City belonged to the B3 subclade, phylogenetic analysis shows that they did not cluster with the strains that caused the 2016 epidemics in the United States and Europe (∼98.21% similarity) or the strains reported in Europe (France) in 2018 (∼97.93% similarity), which also are in subclade B3 (Fig. 1). The similarity between the 2018 Kansas City EV-D68 strains and the 2014 outbreak B1 subclade was ∼94.83%. These are the first publicly available EV-D68 genome sequences collected in the United States during the 2018 epidemics. With the increasing number of AFM cases related to EV-D68 infection, our newly reported 2018 EV-D68 strains could help us understand the epidemiological dynamics and clinical implications of EV-D68 in the United States and globally.

### Data availability.

All sequences were submitted to GenBank with accession numbers MK659588 to MK659604. All raw reads were submitted to SRA. The published SRA submission can be found at BioProject accession number PRJNA532464.
